# Structural Plasticity of Dopaminergic Neurons Requires the Activation of the D3R-nAChR Heteromer and the PI3K-ERK1/2/Akt-Induced Expression of c-Fos and p70S6K Signaling Pathway

**DOI:** 10.1007/s12035-022-02748-z

**Published:** 2022-01-19

**Authors:** Veronica Mutti, Federica Bono, Zaira Tomasoni, Leonardo Bontempi, Adele Guglielmi, Silvia Bolognin, Jens C. Schwamborn, Cristina Missale, Chiara Fiorentini

**Affiliations:** 1 Section of Pharmacology, Department of Molecular and Translational Medicine, Division of Pharmacology, University of Brescia, Viale Europa 11, 25123 Brescia, Italy; 2 Luxembourg Centre for Systems Biomedicine (LCSB), University of Luxembourg, L-4362 Belvaux, Luxembourg

**Keywords:** Heterodimer, Dopamine receptors, Nicotine, ERK1/2, Akt, Structural plasticity

## Abstract

We have previously shown that the heteromer composed by the dopamine D3 receptor (D3R) and the nicotinic acetylcholine receptor (nAChR) (D3R-nAChR heteromer) is expressed in dopaminergic neurons, activated by nicotine and represents the molecular unit that, in these neurons, contributes to the modulation of critical events such as structural plasticity and neuroprotection. We now extended this study by investigating the D3R-nAChR heteromer properties using various cell models such as transfected HEK293 cells, primary cultures of mouse dopaminergic neurons and human dopaminergic neurons derived from induced pluripotent stem cells.

We found that the D3R-nAChR heteromer is the molecular effector that transduces the remodeling properties not only associated with nicotine but also with D3R agonist stimulation: neither nAChR nor D3R, in fact, when express as monomers, are able to elicit these effects. Moreover, strong and sustained activation of the PI3K-ERK1/2**/**Akt pathways is coupled with D3R-nAChR heteromer stimulation, leading to the expression of the immediate-early gene c-Fos and to sustained phosphorylation of cytosolic p70 ribosomal S6 kinase (p70S6K), critical for dendritic remodeling. By contrast, while D3R stimulation results in rapid and transient activation of both Erk1/2 and Akt, that is PI3K-dependent, stimulation of nAChR is associated with persistent activation of Erk1/2 and Akt, in a PI3K-independent way. Thus, the D3R-nAChR heteromer and its ability to trigger the PI3K-ERK1/2/Akt signaling pathways may represent a novel target for preserving dopaminergic neurons healthy and for conferring neuronal protection against injuries.

## Introduction

The dopaminergic (DA) system modulates various physiological functions such as motor activity, cognition, reward, working memory and learning. Consequently, multiple human diseases are associated with DA neurons’ dysfunctions. In particular, Parkinson’s disease (PD), a progressive neurological disorder that mainly affects voluntary movements, is caused by the selective neurodegeneration of DA neurons in the substantia nigra pars compacta; the molecular basis underlying this pathology is not completely understood and curative drugs that may protect DA neurons from death are not yet available. Therefore, a comprehensive analysis of the mechanisms that might preserve DA neurons’ health is crucial for designing strategies to counteract neurodegeneration. Different receptor systems, including the DA D3 receptor (D3R) and the nicotinic acetylcholine receptor (nAChR), have been shown to provide neurotrophic and neuroprotective support to DA neurons [1, 2]. In particular, we have recently identified a receptor heteromer composed of the D3R and the nAChR (D3R-nAChR) as the molecular unit that is highly expressed in DA neurons, is activated by nicotine, and contributes to the modulation of critical events in DA neurons such as structural plasticity and neuroprotection [2–4]. The D3R-nAChR heteromer, first identified in transfected HEK293 cells by BRET, originates from the direct interaction between the D3R and the beta2 subunit of the nAChR [3]. The physiological expression of the D3R-nAChR heteromer has been disclosed by using proximity litigation assay in both mouse DA neurons and human DA neurons derived from induced pluripotent stem cells (iPSCs) [3, 4].

Functionally, D3R-nAChR heteromer activation by nicotine promotes DA neuron structural remodeling including increased dendritic arborization and soma area [3]. The unique involvement of the D3R-nAChR complex in these effects was demonstrated by using specific interfering peptides that prevent the interaction between D3R and nAChR [3]; in these conditions, nicotine-induced neurotrophic effects on DA neurons were, in fact, completely lost [3]. More recently, nicotine activation of the D3R-nAChR heteromer has been associated with the protection of DA neurons from neurotoxicity induced by glucose deprivation [4].

In this study, we further investigated the properties of the D3R-nAChR heteromer, by focusing on the specific role played by the D3R protomer within the heteromer and by analyzing the intracellular signaling pathways activated by D3R-nAChR stimulation. Previous studies suggested that the phosphoinositide-3-kinase–protein kinase (PI3K)-ERK1/2/Akt signaling cascade, likely associated with the D3R [5, 6], could contribute to nicotine-induced remodeling in DA neurons [4, 6]. Therefore, we considered the possibility that the D3R-nAChR heteromer activated by nicotine might signal through the PI3K-ERK1/2/Akt cascade, recruited by the D3R protomer. To address this hypothesis we used different cell models: (1) HEK293 cells either individually expressing or co-expressing the D3R and the nAChR [3]; (2) primary cultures of mouse DA neurons, physiologically expressing the D3R-nAChR heteromer [4], and primary cultures of DA neurons derived from D3R knock out (D3R-KO) mice only expressing the nAChR; 3) human iPSCs-derived DA neurons where the D3R-nAChR complex has been identified by the proximity ligation assay (PLA) [4].

The major finding of this study is that in DA neurons, the D3R-nAChR heteromer is the molecular effector that transduces the neurotrophic effects not only of nicotine but also of D3R agonists. Neither nAChR nor D3R when individually expressed is in fact able to elicit these effects. Moreover, a special transductional feature of the D3R-nAChR heteromer is represented by the persistent activation of the ERK1/2 and Akt signaling that requires PI3K. In addition, the expression of the immediate-early gene c-Fos [7] and the activation of the p70 ribosomal S6 kinase (p70S6K), an enzyme that acts downstream of mTOR pathway [8, 9], are two molecular signals associated with the heteromer and crucially required for DA neuron dendritic remodeling.

## Materials and Methods

### Materials

Human embryonic kidney (HEK) 293 cells were provided by Open Biosystems. Tissue culture medium and fetal bovine serum (FBS) were purchased from Euroclone (Milan, Italy). LY294002, quinpirole, and nicotine were purchased from Tocris (Bristol, UK).

Human mutant pcDNA3-alpha4, pcDNA3-beta2 vectors were kindly provided by Dr. S. Fucile (Sapienza, Università di Roma). GFP-DRD3 was a gift from Jean-Michel Arrang (Addgene plasmid # 24,098; http://n2t.net/addgene:24098; RRID: Addgene_24098) [10]. Cell permeable interfering TAT peptides TAT-D3R (NH2 -YGRKKRRQRRRLKQRRRKRIL-COOH) and the TAT-D3R-Sc scrambled sequence (NH2-YGRKKRRQRRRIRKLRLRQRK-COOH) were purchased from GenScript, Piscataway, USA [3].

### Animals

Dopamine D3 receptor knock out (D3R-KO) mice and their syngeneic wild-type mice (C57BL6/J), used as a control, were obtained from Jackson Laboratory (Bar Harbor, ME) (B6.129S4-Drd3Tm1Dac/J). Animals were bred and housed in the animal house facility of the University of Brescia with water and food ad libitum and a 12-h light–dark cycle. Animals were cared for and killed according to the 2010/63/EU Directive and in conformity to the National Research Guide for the Care and Use of Laboratory Animals. The Animal Research Ethical Committee of the University of Brescia also approved all the procedures. All efforts were made to minimize animal suffering and to reduce the number of animals used.

### Primary Cultures of Mouse Midbrain Neurons

Primary cultures of midbrain neurons were prepared from both wild-type and D3R-KO mice, by dissecting the ventral mesencephalon from E12.5 mouse embryos, as previously described [3, 4]. Embryos were then collected and mechanically dissociated at room temperature, and suspended in Neurobasal medium (Gibco, Invitrogen, Carlsbad, CA, USA) containing 2 mM glutamine and B27 supplement (Gibco, Invitrogen). Cells were seeded on poly-D-lysine/laminin-coated plates (1 × 10^5^ cells/well) or coverslips (8 × 10^4^ cells) and cultured in Neurobasal medium (Gibco, Invitrogen, Carlsbad, CA, USA) containing 2 mM glutamine and 2% B27 supplement (Gibco, Invitrogen) at 37 °C in a humidified 5% CO2 atmosphere. Half of the medium was changed every 2 days until treatment. All pharmacological treatments were performed after 7 days from seeding.

### HEK293 Cell Cultures and Transfection

Human embryonic kidney 293 T (HEK293) cells were cultured in Dulbecco's modified Eagle's medium (DMEM) containing 10% FBS, 2 mM glutamine, 0.1 mM nonessential amino acids, 1 mM sodium pyruvate, 100 U/ml penicillin, and 100 μg/ml streptomycin (all purchased from Euro Clone, Milan, Italy) at 37 °C in an atmosphere of 5% CO2. Cells were transiently transfected with the GFP-DRD3 dopamine vector (D3R) (HEK-D3R cells) (1 μg) or with the pcDNA-alpha4 (1 μg) and pcDNA-beta2 (1 μg) nAChR subunits (HEK-nAChR cells) or co-transfected with both plasmids (HEK- D3R-nAChR cells) using the Arrest-IN reagent (Thermo Scientific), according to the manufacturer’s instructions. The pCDNA3 vector was used to equilibrate the total amounts (2 ug) of transfected DNA.

### Human iPSCs-Derived DA Neuron Differentiation

Human iPSCs from a healthy control were generated and fully characterized by Reinhardt et al., (2013) [11]. Informed consent was obtained prior to cell donation [11]. Mature neurons were obtained by using a dual-SMAD inhibition protocol [12] with some modifications, as previously described [13]. Following 50 days of differentiation, iPSCs-derived neuronal cultures containing ∼40% of DA neurons expressing the DAergic marker tyrosine hydroxylase (TH) enzyme were obtained and used for subsequent treatments and experiments.

### Pharmacological Treatments

For morphological studies, mouse cultures and human iPSCs-derived neurons were treated with nicotine (10 μM) or quinpirole (10 μM) for 72 h (h) in the presence or absence of TAT-D3R (1 μM) peptide or TAT-D3Rsc (1 μM), added 30 min (min) before nicotine or quinpirole incubation and every 24 h until the end of treatment. Cells were then fixed and analyzed by immunocytochemistry for morphometric parameters, as described below.

In another set of experiments, mouse cultures and human iPSCs-derived neurons were treated with nicotine (0.01 μM) or quinpirole (1 μM) or nicotine (0.01 μM) plus quinpirole (1 μM) for 72 h and morphologically analyzed by immunocytochemistry, as described below.

For biochemical analysis, HEK-nAChR, HEK-D3R, and HEK-D3R-nAChR cells were used 24 h post-transfection, maintained in serum-free medium for 16 h and treated. In particular, HEK-nAChR cells were treated with nicotine (10 μM) for different times (5–60 min) or with nicotine (10 μM; 30 min) in the presence or in the absence of the PI3K inhibitor LY294002 (10 μM), added 30 min before nicotine stimulation. In parallel, HEK-D3R cells were treated with quinpirole (10 μM) for different times (0–60 min) or with quinpirole (10 μM; 30 min), in the presence or in the absence of LY294002 (10 μM), added 30 min before quinpirole stimulation. Finally, HEK- D3R-nAChR cells were treated with nicotine (10 μM) or quinpirole (10 μM) for different times (5–60 min) and with nicotine (10 μM; 30 min) or quinpirole (10 μM; 30 min) in the presence or in the absence of LY2940022 (10 μM), added 30 min before nicotine or quinpirole stimulation. Cells were analyzed for ERK1/2 phosphorylation (pERK1/2) and Akt phosphorylation at Thr308 (pAkt) by western blot, as described below. As a control, HEK-nAChR cells were incubated with quinpirole (10 μM) for different times (0–60 min).

In another set of experiments, primary cultures of mouse midbrain neurons were treated with nicotine (10 μM) or quinpirole (10 μM) for different times (5–14 h) and analyzed for pERK1/2 by both western blot and immunofluorescence, as described below. Moreover, mouse cultures were treated with nicotine (10 μM; 30 min) or quinpirole (10 μM; 30 min) in the presence or in the absence of TAT-D3R (1 μM) peptide, TAT-D3Rsc (1 μM) or LY2940022 (10 μM) and analyzed for pERK1/2 and pAkt by western blot. In parallel experiments, primary cultures of midbrain neurons were obtained from D3R-KO mice and treated with nicotine (10 μM) for different times (5–60 min) and analyzed for pERK1/2 by both western blot and immunofluorescence.

Moreover, primary cultures of midbrain neurons obtained from D3R-KO mice were treated with nicotine (10 μm; 30 min) in the presence or in the absence of the PI3K inhibitor LY2940022 (10 μM) and analyzed for pERK1/2 and pAkt by western blot.

Finally, neuronal culture derived from both wild-type and D3R-KO mice was treated with nicotine or quinpirole (both at 10 μM; 5–14 h) or with nicotine (10 μM; 5–14 h), respectively) and analyzed for p70S6K phosphorylation (p-p70S6K), CREB phosphorylation (pCREB), and c-Fos levels by western blot.

### Immunocytochemistry and Morphometric Analyses

Neurons were fixed using 4% PFA for 10 min and incubated in 3% hydrogen peroxidase for 10 min to inhibit endogenous peroxidase activity. Cells were then blocked in PBS containing 0.1% Triton x-100 (Sigma-Aldrich) and 5% BSA and incubated with the rabbit anti-TH polyclonal antibody (1:700, Santa Cruz) at 4 °C overnight. The next day, cells were incubated with the biotinylated anti-rabbit antibody (1:350; Jackson Immuno Research) for 30 min at room temperature and incubated with avidin–biotin horseradish peroxidase complex. Staining with peroxidase was performed by incubation in PBS containing 1% 3–3′ diaminobenzidine and 0.01% H_2_O_2_ (Sigma-Aldrich). Digital images of the immunocytochemical assays were captured with an Olympus IX51 microscope connected to an Olympus digital camera and analyzed using the ImageJ software (National Institutes of Health, Bethesda, MD, USA). The morphologic indicators of structural plasticity were as follows: (1) maximal dendrite length, defined as the distance from the soma to the tip of the longest dendrite for each neuron; (2) primary dendrites numbers, defined as those directly stemming from the soma; (3) soma area, assessed by measuring the surface (µm^2^) included by the external perimeter drawn on the cell membrane of neurons identified by TH staining. Positive neurons were analyzed by a blinded examiner using a × 20 objective (Olympus IX51). Three slides per treatment group were examined and more than fifty frames for each coverslip were analyzed to obtain measurements from at least 30 TH-positive neurons.

### Immunofluorescence

Human iPSCs-derived DA neurons were fixed in phosphate-buffered saline (PBS) containing 3% paraformaldehyde (Sigma-Aldrich)⁄3% sucrose (Sigma-Aldrich) and blocked in PBS containing 0.1% Triton X-100 (Promega, Madison, WI, USA), 5% bovine serum albumin (BSA; Sigma-Aldrich). Neurons were incubated overnight at 4 °C with the tyrosine hydroxylase (TH, 1:700; Santa Cruz Biotechnology) and the dopamine transporter (DAT, 1:400; Santa Cruz Biotechnology) primary antibody, and then incubated for 30 min at room temperature with appropriate Alexa Fluor 488- or Cy3-conjugated secondary antibodies (Jackson Immuno Research). Nuclei were stained with DAPI.

Mouse primary DA neurons were fixed, blocked in PBS containing 0.1%Triton x-100 (Sigma-Aldrich) and 5% bovine serum albumin (BSA; Sigma-Aldrich), and incubated at 4 °C overnight with the rabbit anti-TH (1:700; Santa Cruz Biotechnology) and with the mouse anti-phospho-p44/42 MAPK (pERK1/2, 1:400; Cell Signaling) primary antibody. Cells were then incubated for 30 min at room temperature with the appropriate Alexa Fluor 488- and Cy3-conjugated secondary antibodies (Jackson Immuno Research). Nuclei were stained with DAPI.

Images from both mouse and human iPSC-derived DA neurons were captured using a Zeiss LSM 880 confocal microscope equipped with Plan-Apochromat 63 × /1.4 numerical aperture oil objective and examined using the ZEN 2.3 software (Carl Zeiss AG, Oberkochen, Germany). The same software was used for the analysis of the pERK1/2/DAPI or pERK1/2/TH co-localization relative area. Analysis of the pERK1/2 intensity was performed by using the ImageJ software (National Institutes of Health, Bethesda, MD, USA). A minimum of 10 fields containing TH-positive neurons for each condition were selected and each experimental condition was performed in triplicate at least three times.

### Proximity Ligation Assay

In situ proximity ligation assay (PLA) was carried out in HEK-D3R-nAChR transfected cells by using the Duolink in situ detection kit (Sigma-Aldrich) according to the manufacturer’s instructions, as previously reported [3, 4]. Briefly, cells were fixed in PBS containing 4% paraformaldehyde (Sigma-Aldrich) for 10 min at room temperature, washed in PBS and blocked with blocking solution (Duolink, Sigma-Aldrich) for 30 min at 37 °C. Cells were next incubated overnight at 4 °C with the goat polyclonal anti-D3R (1:50; Santa Cruz Biotechnologies) and the rabbit monoclonal anti-alpha4 nAChR subunit (1:100; Sigma-Aldrich) antibodies. After washing, cells were incubated with the anti-rat MINUS and the anti-goat PLUS probes (Duolink, Sigma-Aldrich) for 30 min at 37 °C, followed by ligation and amplification reaction using Duolink far red detection reagent. Cells were then mounted using mounting medium containing 4′,6-diamidino-2-phenylindole (DAPI; Duolink, Sigma-Aldrich) and analyzed using a Zeiss LSM 880 confocal microscope equipped with Plan-Apochromat 63 × /1.4 numerical aperture oil objective and examined using the ZEN 2.3 software (Carl Zeiss AG, Oberkochen, Germany).

### Western Blot

Cells were washed with ice-cold PBS and lysed in a buffer containing 50 mM Tris (pH 7.4), 150 mm NaCl, 0.5% sodium deoxycholate, 0.1% sodium dodecyl sulphate, 1% Igepal, 1 mM polymethanesulphonyl fluoride (all from Sigma-Aldrich), and complete protease inhibitors (Roche Diagnostics, Mannhein, Germany); protein concentration was measured with a DC-protein assay (Bio-Rad, Hercules, CA, USA). Aliquots of total proteins were resolved by sodium dodecyl sulphate polyacrylamide gel electrophoresis, blotted onto a PVDF membrane (Immobilon-P; Millipore), and blocked with 5% not-fat milk in 0.1 M Tris-buffered saline (pH = 7.4) for 30 min. Membranes were then incubated with the following primary antibodies: anti-pERK1/2 (1:2500; Santa Cruz Biotechnology), anti-pAkt (Thr308; 1:1000, Immunological Sciences), anti-TH (1:1500; Millipore), anti-pCREB (1:2000, Millipore), anti-c-Fos (1:2000, GeneTex), anti-p-p70S6K (1:1000; Cell Signaling), and anti-alpha-tubulin (1:300,000; Sigma-Aldrich). Blots were incubated with appropriate horseradish peroxidase–conjugated secondary antibodies (Santa Cruz Biotechnology) and signals were detected by enhanced chemiluminescence (ECL) (GeneSpin). The membranes were scanned and analyzed using gel-pro analyzer software (Media Cybernetics, Bethesda, MD, USA).

### Statistical Analysis

Each experiment was repeated at least three times. Values are expressed as mean ± standard error of the mean (SEM). If not stated otherwise, significant differences from control conditions were determined using analysis of variance (ANOVA) followed by Bonferroni’s test for multiple comparisons. For comparison between two groups, an unpaired Student’s *t* test was used. All statistical analyses were performed by GraphPad prism version 4.00 for Windows (GraphPad Software, San Diego, CA, USA). Correlations were assessed by calculating the correlation coefficient between two variables using the same statistical package.

## Results

### D2R/D3R Agonists Exert Neurotrophic Effects in Mouse and Human DA Neurons Acting Through the D3R-nAChR Heteromer

**Fig.1:**
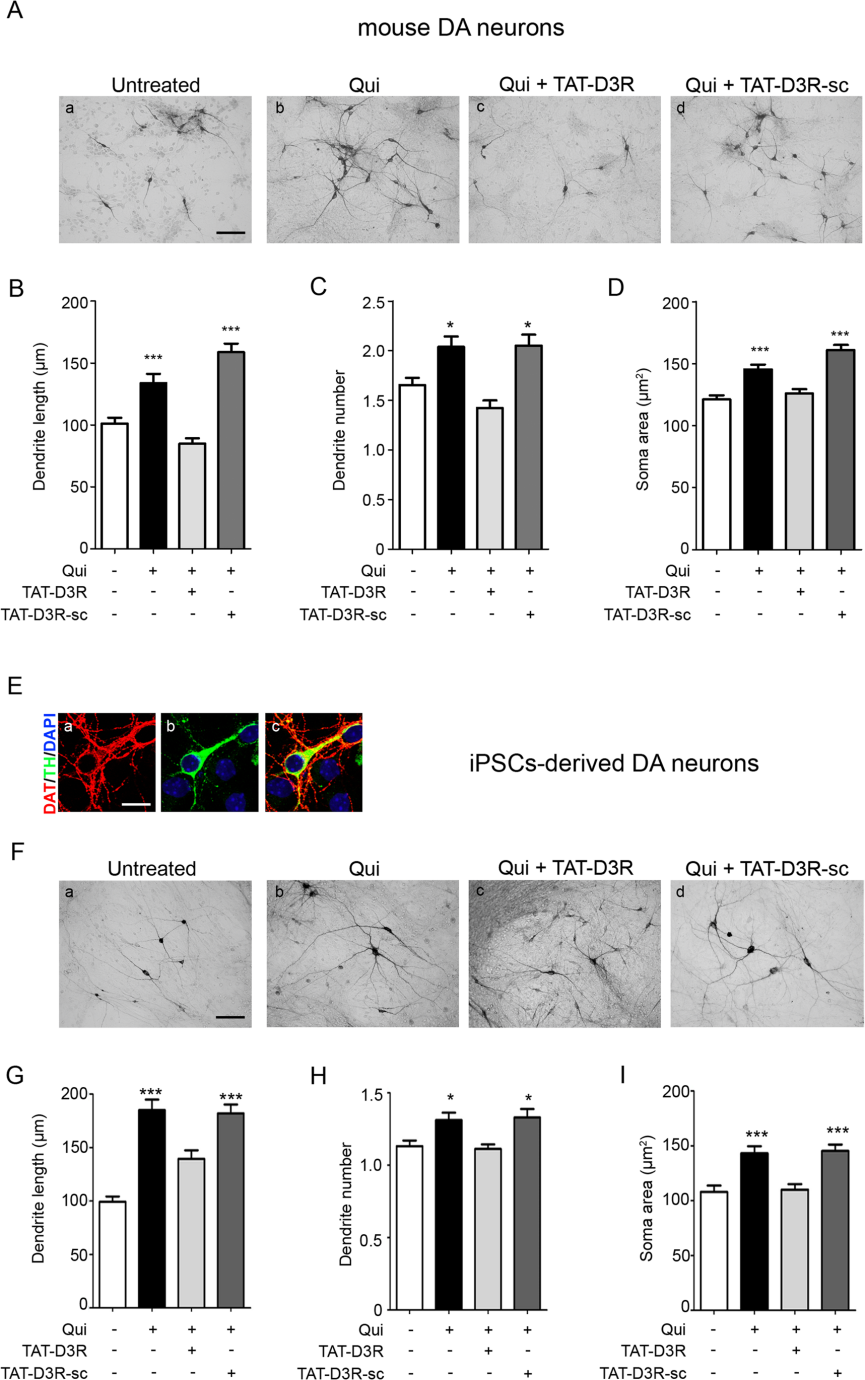
In mouse and human iPSCs-derived DA neurons, quinpirole-induced structural plasticity requires the D3R-nAChR heteromer. **A**–**D** Primary cultures of mouse mesencephalic neurons were exposed to quinpirole (Qui; 10 μM) for 72 h (h) in the absence or in the presence of TAT-D3R (1 μM) or TAT-D3R-sc (1 μM), added as described in the “Materials and Methods” section. **A** Representative microphotographs of untreated (a), quinpirole-(b) and quinpirole/TAT-D3R-(c) or quinpirole/TAT-D3R-sc-treated (d) neurons positive for TH in immunohistochemistry analysis. Scale bar: 100 μm. **B**–**D** Morphologic effects of quinpirole on maximal dendrite length (**B**), number of primary dendrites (**C**), and soma area (**D**), calculated as described the “Materials and Methods” section. Bars represent the mean ± SEM of three independent experiments (***p < 0.001, *p < 0.05 vs untreated; post hoc Bonferroni’s test). **E** Representative images of immunofluorescence analyses of DAT (red, panel a), TH (green, panel b), and co-staining (panel c) in human iPSC-derived DA neurons at day 50 of differentiation. Nuclei were stained with DAPI (blue). Scale bar: 25 μm. **F**–**I** Human iPSCs-derived neurons (day 50) were treated with quinpirole (Qui; 10 μM) for 72 h in the absence or in the presence of TAT-D3R (1 μM) or TAT-D3R-sc (1 μM), added as described the “Materials and Methods” section. **E** Representative microphotographs of neurons treated as described above and positive for TH in immunohistochemistry analysis. Scale bar: 100 μm. **F**–**H** Quantitative analysis of maximal dendrite length (**F**), number of primary dendrites (**G**), and soma area (**H**), calculated as described the “Materials and Methods” section. Bars represent the mean ± SEM of three independent experiments (***p < 0.001, *p < 0.05 vs untreated; post hoc Bonferroni’s test)

We have reported that in mouse DA neurons, chronic activation of the D3R-nAChR heteromer by nicotine exerts neurotrophic effects [3]. To investigate the role of the D3R, primary cultures of mouse mesencephalic neurons, containing 5–10% of DA neurons [3] were stimulated with the D2R/D3R agonist quinpirole (10 μM) for 72 h (h) and analyzed for morphology. As reported in Fig. 1 (panels A–D), D2R/D3R stimulation increased the length of the primary dendrite (panel B), the dendrite number (panel C), and soma area (panel D) of DA neurons, identified by TH staining. Stimulation with quinpirole was also performed in the presence of the TAT-D3R interfering peptide disrupting the interaction between D3R and the beta2 subunit of nAChR [3, 4, 14]. The results show that the TAT-D3R peptide (1 μM), but not its scrambled counterpart (TAT-D3R-Sc; 1 μM), abolished the neurotrophic effects elicited by quinpirole in mouse DA neurons (Fig. 1, panels A–D) suggesting that the remodeling properties of D2R/D3R agonists [5, 15–17] are likely mediated by their interaction with the D3R-nAChR heteromeric complex.

The ability of the D2R/D3R agonists in inducing morphological effects through the D3R-nAChR heteromer was also investigated in human DA neurons derived from iPSCs [4, 13]. iPSC-derived neuronal cultures, containing about 40% of TH-positive neurons also co-expressing dopamine transporter (DAT) (Fig. 1, panel E) [13], were incubated with quinpirole (10 µM, 72 h) and TH-positive neurons were analyzed for morphological remodeling (Fig. 1, panel F–I). Quinpirole induced a significant increase of the maximal length of the primary dendrite (panel G), number of dendrites (panel H), and soma area (panel I), compared to untreated cells. Neurons were then treated with quinpirole in the presence of the TAT-D3R peptide or its corresponding scrambled peptide (both at 1 µM) and the results showed that the remodeling properties of quinpirole were abolished in the presence of the interfering peptide, but not of the scrambled one (Fig. 1, panel F–I). Therefore, as in mouse DA neurons, also in human DA neurons, the D3R-nAChR heteromer is the receptor unit that transduces the neurotrophic effects of both nicotine and D3R agonists. Interestingly, in DA neurons that co-express D2R and D3R, counteracting heterodimer formation prevented quinpirole from inducing morphological changes, thus excluding the involvement not only of the D3R monomer but also of the D2 receptors (D2R).

### Combining Ineffective Doses of Nicotine and Quinpirole Exerts Neurotrophic Effects on Mouse and Human DA Neurons

**Fig. 2:**
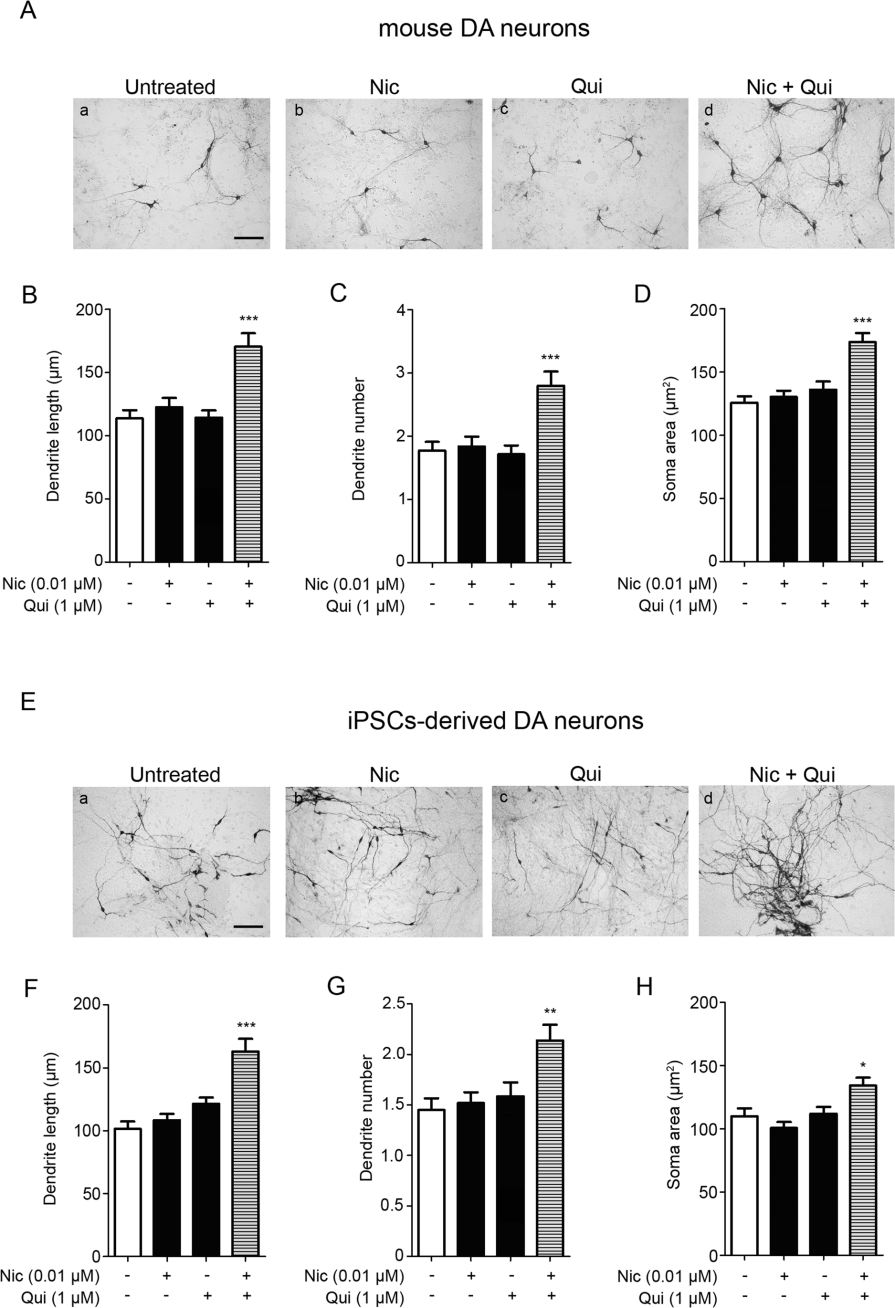
In mouse and human iPSCs-derived DA neurons, the combination of ineffective doses of nicotine and quinpirole exerts neurotrophic effects. **A**–**D** Primary cultures of mouse mesencephalic neurons were exposed to nicotine (Nic; 0.01 μM) or quinpirole (Qui; 1 μM) or nicotine (Nic; 0.01 μM) plus quinpirole (Qui; 1 μM) for 72 h. **A** Representative microphotographs of untreated (a), nicotine-(b), quinpirole-(c), or nicotine plus quinpirole-treated (d) neurons positive for TH in immunohistochemistry analysis. Scale bar: 100 μm. **B**–**D** Quantitative analysis of maximal dendrite length (**B**), number of primary dendrites (**C**), and soma area (**D**), calculated as described the “Materials and Methods” section. Bars represent the mean ± SEM of three independent experiments (***p < 0.001 vs untreated; post hoc Bonferroni’s test). **E**–**H** Human iPSCs-derived neurons (day 50) were treated with nicotine (Nic; 0.01 μM) or quinpirole (Qui; 1 μM) or nicotine (Nic; 0.01 μM) plus quinpirole (Qui; 1 μM) for 72 h. **E** Representative microphotographs of untreated (a), nicotine-(b), quinpirole-(c), or nicotine plus quinpirole-treated (d) neurons positive for TH in immunohistochemistry analysis. Scale bar: 100 μm. **F**–**H** Quantitative analysis of maximal dendrite length (**F**), number of primary dendrites (**G**), and soma area (**H**), calculated as described the “Materials and Methods” section. Bars represent the mean ± SEM of three independent experiments (***p < 0.001, **p < 0.01, *p < 0.05 vs untreated; post hoc Bonferroni’s test)

To evaluate the possible existence of a combinatorial effect of nicotine and quinpirole in inducing DA neuron remodeling mouse (Fig. 2, panel A–D) and human (Fig. 2, panel E–H) neurons were treated with nicotine (0,01 μM), quinpirole (1 μM), and a combination of nicotine and quinpirole (0,01 and 1 μM, respectively) for 72 h; as reported in Fig. 2 and according to previous studies [5, 6], at low doses, neither nicotine nor quinpirole was able to exert morphological changes. However, analyses of TH-positive mouse and human neurons showed that the simultaneous treatment with low doses of both compounds significantly increased the maximal length of the primary dendrite (panel B and F), the number of dendrites (panel C and G), and the soma area (panel D and H).

### Stimulation of the D3R-nAChR Heteromer Induces Persistent Phosphorylation of ERK1/2 that Requires PI3K Activation

**Fig. 3:**
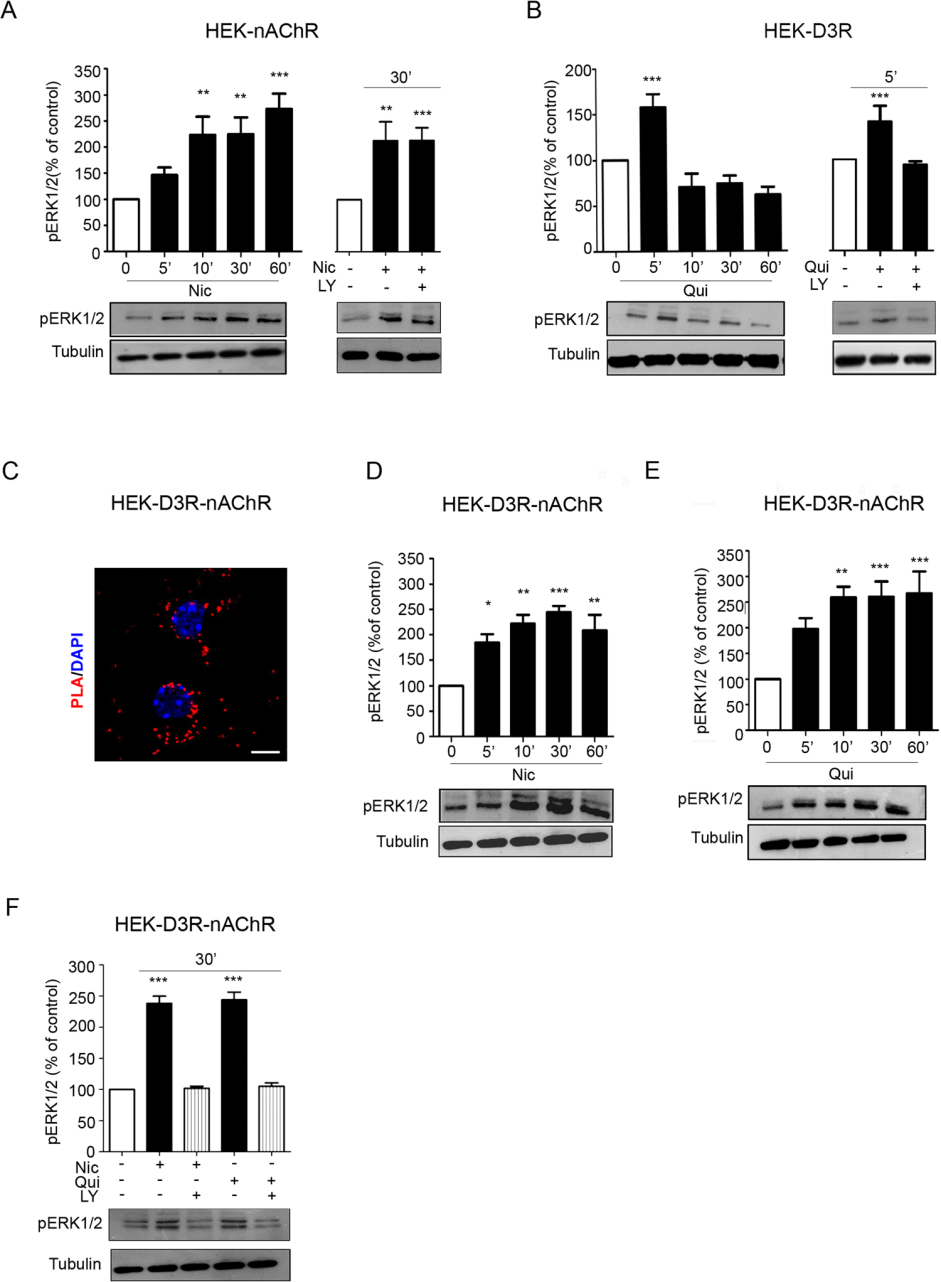
Activation of ERK1/2 induced by stimulation of D3R, nAChR, and D3R/nAChR heteromer in HEK293 cells. **A** HEK293T cells transiently expressing the alpha4beta2 nAChR subunit (HEK-nAChR) were stimulated with nicotine (Nic; 10 μM) for different time (5–60 min) and analyzed for ERK1/2 phosphorylation (pERK1/2) by western blot; moreover, HEK-nAChR cells were stimulated with nicotine (10 μM) for 30 min in the presence or in the absence of LY294002 (LY; 10 μM) and analyzed for pERK1/2. Upper panel, densitometric analysis of blots (*n* = 3) with specific levels of pERK1/2 normalized to the corresponding tubulin level. Lower panel, representative blot (image) of pERK1/2; **B** HEK293T cells transiently expressing the D3R (HEK-D3R) were stimulated with quinpirole (Qui, 10 μM) for different times (5–60 min) and analyzed for pERK1/2 by western blot; moreover, HEK-D3R cells were stimulated with quinpirole (10 μM) for 30 min in the presence or in the absence of LY294002 (10 μM) and analyzed for pERK1/2. Upper panel, densitometric analysis of blots (*n* = 3) with specific levels of pERK1/2 normalized to the corresponding tubulin level. Lower panel, representative blot of pERK1/2 analyses. **C** Analyses of HEK293T cells transiently expressing both D3R and alpha4beta2 nAChR (HEK-D3R-nAChR) by PLA (red spot). Nuclei are detected with DAPI (blue). Scale bar = 20 μm. **D**–**E** HEK-D3R-nAChR were stimulated with nicotine (10 μM) (**D**) or quinpirole (10 μM) (**E**) for different times (5–60 min). Upper panel, densitometric analysis of blots (*n* = 3) with specific levels of pERK1/2 normalized to the corresponding tubulin level. Lower panel, representative pERK1/2 phosphorylation analyses by western blot. **F** HEK-D3R-nAChR cells were stimulated with nicotine (10 μM) or quinpirole (10 μM) for 30 min in the presence or in the absence of LY294002 (10 μM) and analyzed for pERK1/2. Upper panel, densitometric analysis of blots (*n* = 3) with specific levels of pERK1/2 normalized to the corresponding tubulin levels. Lower panel, representative image of pERK1/2. Data were statistically analyzed by one-way ANOVA followed by post hoc comparison with Bonferroni test. (***p < 0.001, **p < 0.01, *p < 0.05 vs untreated; post hoc Bonferroni’s test)

In mouse DA neurons, the engagement of the PI3K-ERK1/2 pathway is required for nicotine-induced structural plasticity [6], suggesting that ERK1/2 pathway activation may be associated with D3R-nAChR heteromer stimulation and strictly related to its ability in exerting neurotrophic effects. On these bases, the effects of D3R-nAChR heteromer stimulation on ERK1/2 signaling were investigated. HEK293 cells transiently expressing alpha4beta2-nAChR (HEK-nAChR) or D3R (HEK-D3R) or co-expressing D3R and alpha4beta2-nAChR (HEK-D3R-nAChR) were used. HEK-nAChR cells were stimulated with nicotine (10 μM) for 5–60 min (min) and analyzed for ERK1/2 phosphorylation (pERK1/2) by western blot. As reported in Fig. 3 (panel A), starting from 5 min of stimulation, nicotine elicited a persistent increase of the pERK1/2 levels, which were still elevated after a 60-min stimulation. The role of PI3K was investigated by using the specific inhibitor LY294002. HEK-nAChR cells were stimulated with nicotine (10 μM) for 30 min in the presence or in the absence of LY294002 (10 μM) and analyzed for pERK1/2. As shown in Fig. 3 (panel A), inhibiting PI3K did not impair nicotine-mediated ERK1/2 phosphorylation, suggesting that PI3K is not involved in nAChR signaling.

Activation of ERK1/2 was analyzed in HEK-D3R cells incubated with quinpirole (10 μM) for 5–60 min. According to previous data [5, 18, 19], quinpirole stimulation of the D3R induced rapid and transient activation of ERK1/2, with a phosphorylation peak at 5 min, which rapidly decreased toward basal levels; moreover, the PI3K inhibitor LY294002 (10 μM) abolished D3R-mediated ERK1/2 phosphorylation (Fig. 3, panel B). As a control, HEK-nAChR cells were also incubated in the presence of quinpirole (10 µM; 0–60 min) and analyzed for Erk1/2 activation, showing its inability to activate the nAChR receptor (data not shown).

HEK-D3R-nAChR cells were treated with nicotine (10 μM) or quinpirole (10 μM) for 5–60 min and analyzed for pERK1/2. In these cells, the ability of D3R to interact with the nAChR, previously shown by BRET assay (Bontempi et al., 2017), was also demonstrated by using the anti-D3R and the anti-alpha4 nAChR subunit antibodies in PLA experiments (Fig. 3, panel C). As shown in Fig. 3 (panels D and E), both nicotine and quinpirole increased pERK1/2 levels in a long-lasting way; these effects were abolished by co-incubation with LY294002 (10 μM; 30 min, panel F), suggesting that the persistent activation of ERK1/2 by the D3R-nAChR heteromer requires PI3K activation.

**Fig. 4:**
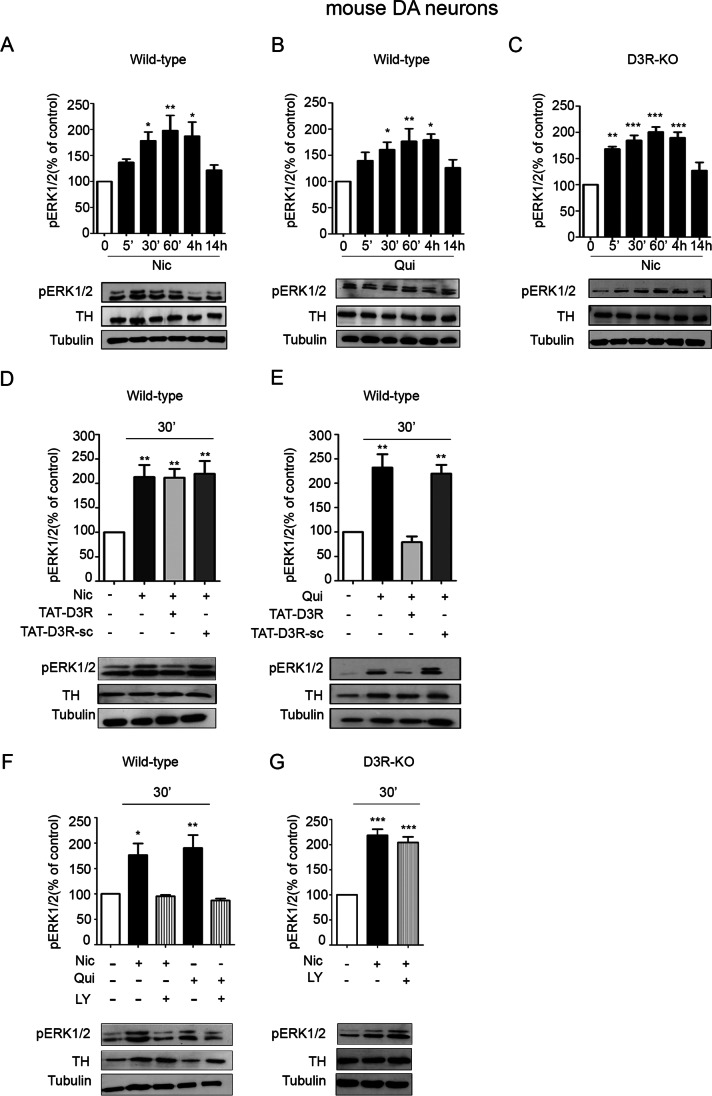
In mouse DA neurons, nicotine- and quinpirole-induced activation of ERK1/2 requires the D3R-nAChR heteromer. **A** Primary cultures of mouse mesencephalic neurons were stimulated with nicotine (Nic; 10 μM) for different times (0–14 h) and analyzed for pERK1/2 by western blot. Upper panel, densitometric analysis of blots (*n* = 3) with specific levels of pERK1/2 normalized to the corresponding TH and tubulin levels. Lower panel, representative blot of pERK1/2. (B) Primary cultures of mouse mesencephalic neurons were stimulated with quinpirole (Qui; 10 μM) for different times (0–14 h) and analyzed for pERK1/2 by western blot. Upper panel, densitometric analysis of blots (*n* = 3) with specific levels of pERK1/2 normalized to the corresponding TH and tubulin levels. Lower panel, representative blot of pERK1/2. **C** Primary cultures of mouse mesencephalic neurons derived from D3R knock out (D3R-KO) mice were stimulated with nicotine (10 μM) for different times (0–14 h) and analyzed for pERK1/2 by western blot. Upper panel, densitometric analysis of blots (*n* = 3) with specific levels of pERK1/2 normalized to the corresponding TH and tubulin levels. Lower panel, representative blot of pERK1/2. **D**–**E** Primary cultures of mouse mesencephalic neurons were exposed to nicotine (10 μM) (**D**) or quinpirole (10 μM) (**E**) for 30 min in the presence or in the absence of TAT-D3R (1 μM) or TAT-D3R-sc (1 μM) and analyzed for pERK1/2 by western blot. Upper panel, densitometric analysis of blots (*n* = 3) with specific levels of pERK1/2 normalized to the corresponding TH and tubulin levels. Lower panel, representative blot of pERK1/2. **F** Primary cultures of mouse mesencephalic neurons were exposed to nicotine (10 μM) or quinpirole (10 μM) for 30 min in the presence or in the absence of LY294002 (LY; 10 μM) and analyzed for pERK1/2. Upper panel, densitometric analysis of blots (*n* = 3) with specific levels of pERK1/2 normalized to the corresponding TH and tubulin levels. Lower panel, representative image of pERK1/2. **G** Primary cultures of mouse mesencephalic neurons derived from D3R-KO mice were exposed to nicotine (10 μM) for 30 min in the presence or in the absence of LY294002 (10 μM) and analyzed for pERK1/2. Upper panel, densitometric analysis of blots (*n* = 3) with specific levels of pERK1/2 normalized to the corresponding TH and tubulin levels. Lower panel, representative image of pERK1/2. Data were statistically analyzed by one-way ANOVA followed by post hoc comparison with Bonferroni test. (***p < 0.001, **p < 0.01 *p < 0.05 vs untreated; post hoc Bonferroni’s test)

The activation of the PI3K-ERK1/2 pathway by the D3R-nAChR heteromer was analyzed in primary cultures of mouse mesencephalic neurons derived from both wild-type and D3R knock-out mice (D3R-KO) (Fig. 4). Analysis of ERK1/2 phosphorylation in wild-type cultures, treated either with nicotine (10 μM) or quinpirole (10 μM) for different times (5 min–14 h), showed that both compounds activated ERK1/2 in a persistent way; pERK1/2 levels were, in fact, significantly elevated between 30 min and 4 h of stimulation, while at 14 h post-treatment, pERK1/2 levels were comparable to that of basal levels (Fig. 4, panel A and B). According to the data obtained in HEK-nAChR cells, in cultures derived from D3R-KO mice, only expressing the nAChR, treatment with nicotine for different times (10 μM; 5 min–14 h) produced a persistent activation of ERK1/2, from 5 min up to 4 h (Fig. 4, panel C).

To associate the activation of ERK1/2 pathway with the D3R-nAChR heteromer, neuronal cultures from wild-type mice were treated either with nicotine (10 μM) or quinpirole (10 μM) for 30 min both in the presence and in the absence of the TAT-D3R or TAT-D3R-sc interfering peptides (1 μM) (Fig. 4, panels D and E). As expected, since at this time, the activation of both D3R-nAChR heteromer and nAChR led to persistent phosphorylation of ERK1/2, nicotine-induced long-lasting pERK1/2 activation was unaffected by the disruption of the D3R-nAChR heteromer (Fig. 4, panel D). By contrast, as in HEK-D3R cells, in the presence of the TAT-D3R peptide (1 μM), quinpirole stimulation of D3R, no longer interacting with the nAChR, did not produce persistent ERK1/2 phosphorylation (Fig. 4, panel E). As shown for HEK-293 cells co-expressing both the D3R and the nAChR, treating neuronal cultures from wild-type mice with nicotine or quinpirole (both at 10 μM) for 30 min in the presence of the PI3K inhibitor LY294002 (10 um), significantly decreased pERK1/2 levels (Fig. 4, panel F). Interestingly, in neurons from D3R-KO mice treated for 30 min with nicotine (10 μM), the presence of the PI3K inhibitor LY294002 (10 μM) did not affect ERK1/2 phosphorylation (Fig. 4, panel G). Together, these data suggest that stimulation of the D3R-nAChR heteromer in DA neurons results in the persistent activation of ERK1/2, an effect requiring PI3K activation. By contrast, nAChR-induced prolonged activation of ERKs is PI3K-independent.

### PI3K-ERK1/2 Activation Induced by Nicotine and Quinpirole Modulates Cytoplasmic p70S6K and Increases Nuclear c-Fos Levels in Mouse DA Neurons

**Fig. 5:**
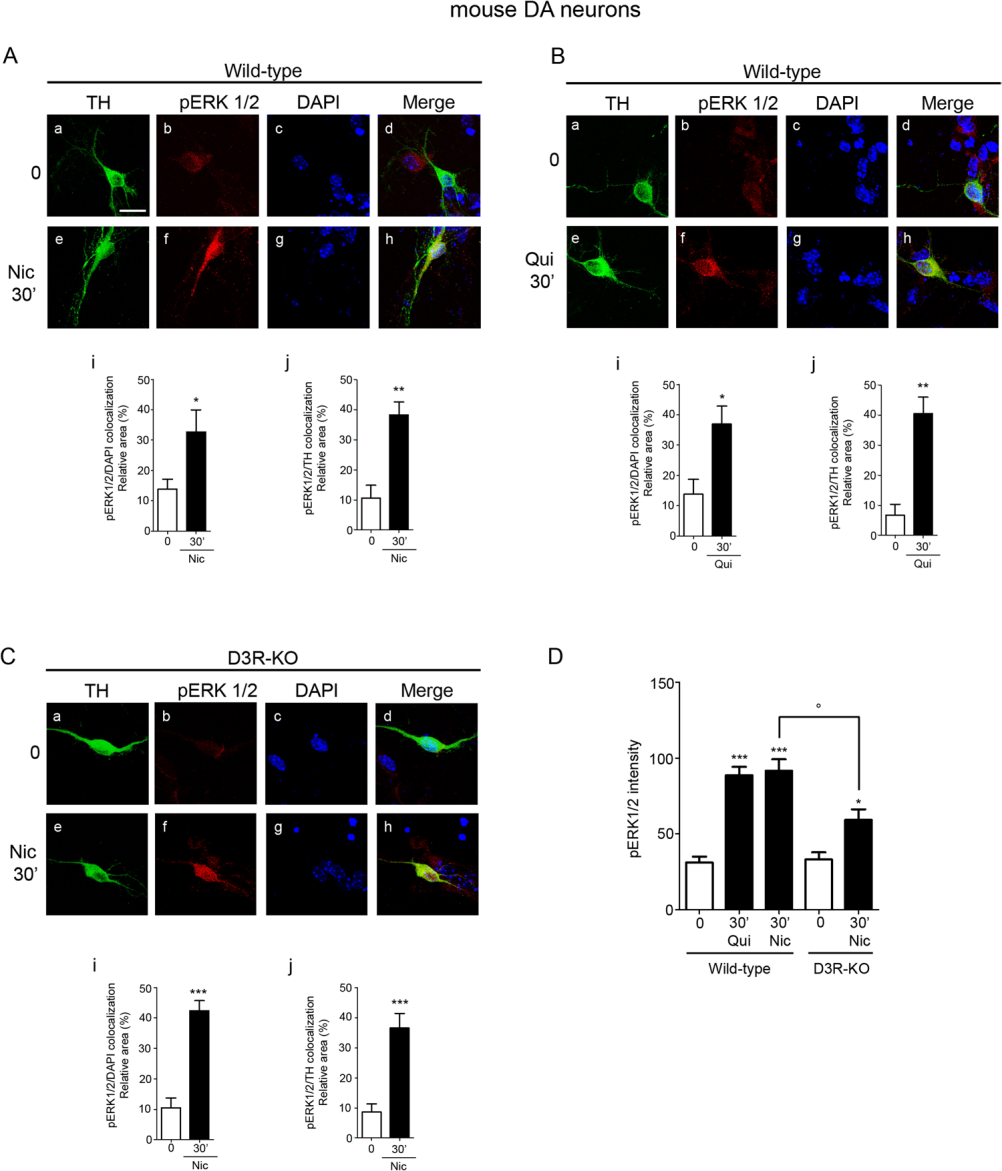
Analyses of pERK1/2 localization and strength induced by nicotine and quinpirole in mouse DA neurons from wild-type and D3R-KO mice. **A**–**B** Primary cultures of mouse mesencephalic neurons derived from wild-type mice were exposed to nicotine (Nic; 10 μM) (**A**) or quinpirole (Qui; 10 μM) (**B**) for 30 min and analyzed for pERK1/2 by immunocytochemistry and confocal analysis. Representative microphotographs of untreated (a–d) and nicotine-treated or quinpirole-treated (e–h) neurons positive for TH in confocal analysis. Quantification of the pERK1/2/DAPI (i) or pERK1/2/TH (j) co-localization relative area. **C** Primary cultures of mouse mesencephalic neurons derived from D3R-KO mice were exposed to nicotine (10 μM) for 30 min and analyzed for pERK1/2 by immunocytochemistry and confocal analysis. Representative microphotographs of untreated (a–d) and nicotine-treated neurons positive for TH in confocal analysis. Quantification of the pERK1/2/DAPI (i) or pERK1/2/TH (j) co-localization relative area. (TH, green; pERK1/2, red; nuclei stained with DAPI). Scale bar: 20 μM. Bars represent the mean ± SEM of three independent experiments (***p < 0.001, **p < 0.01, *p < 0.05 vs untreated; post hoc Bonferroni’s test). **D** Quantification of the pERK1/2 intensity in TH positive-neurons from wild-type and D3R-KO mice (***p < 0.001, *p < 0.05 vs untreated; post hoc Bonferroni’s test; °*p* < 0.05 vs wild-type; Student’s *t test*)

It is well known that pERK1/2 can modulate the activity of many substrates localized both in the cytoplasm and in the nucleus, thus determining distinct biological outcomes [20]. Therefore, the cellular distribution of D3R-nAChR heteromer-induced pERK1/2 was investigated. Neuronal cultures from wild-type mice were treated with nicotine or quinpirole (both at 10 μM) for 30 min and TH-positive neurons were analyzed by immunofluorescence. pERK1/2 co-staining with the nuclear marker DAPI was used for quantifying pERK1/2 into nuclei, while co-staining with TH was used for cytoplasmic pERK1/2 quantification. As shown in Fig. 5, pERK1/2 induced by nicotine (panel A) and quinpirole (panel B) were detected in both cytoplasm and nucleus. Similar results were obtained in cultures from D3R-KO mice (Fig. 5, panel C) where incubation with nicotine (10 μM; 30 min) led to pERK1/2 localization both at cytoplasmic and nuclear sites. However, quantification of pERK1/2 signals in TH-positive neurons, measured by using immunofluorescence, showed that pERK1/2 activated by either nicotine or quinpirole in wild-type DA neurons was quantitatively and significantly higher than activated by nicotine in D3R-KO DA neurons (Fig. 5, panel D).

**Fig. 6:**
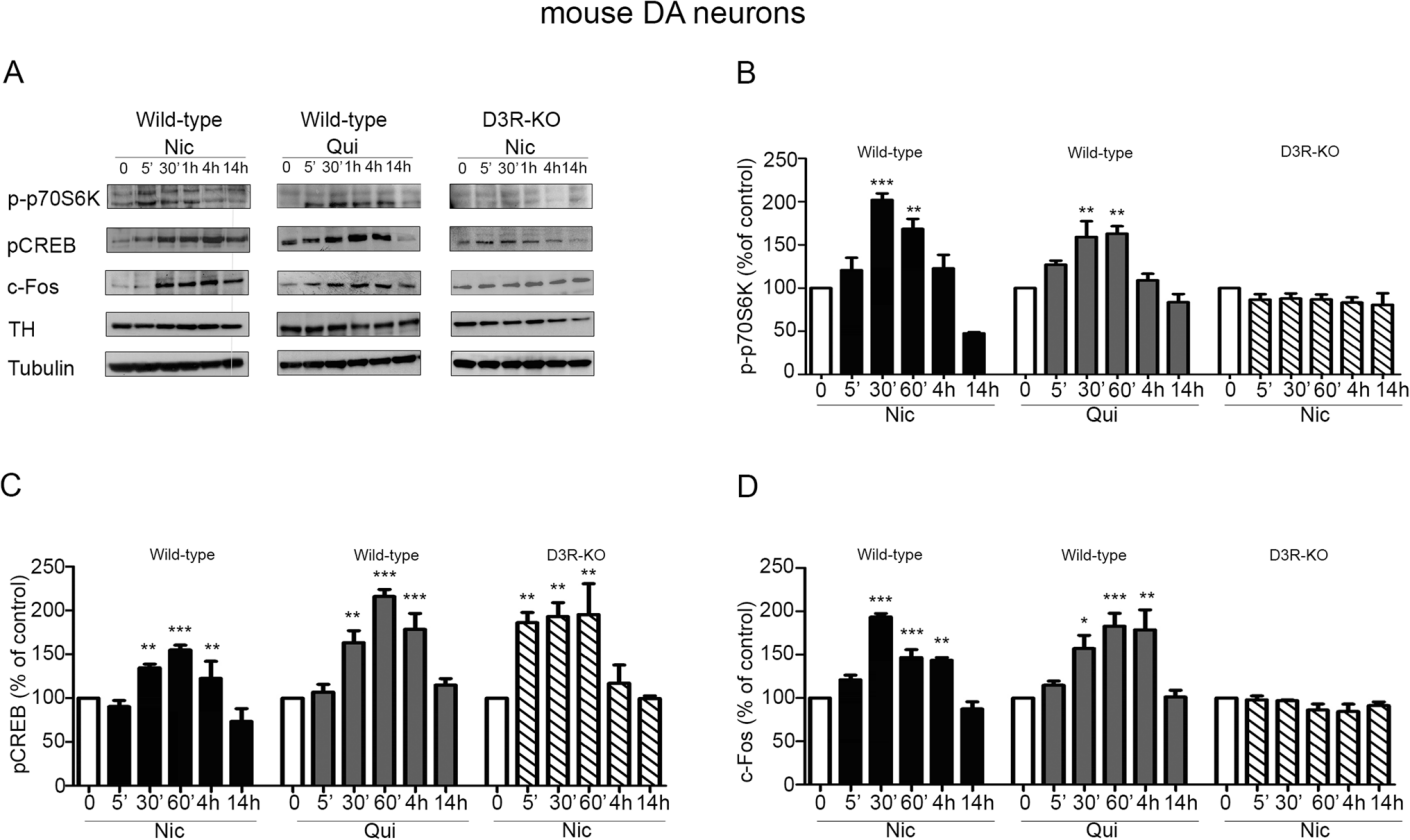
Analyses of p70S6K phosphorylation, CREB phosphorylation and c-Fos levels induced by nicotine and quinpirole in mouse neuronal cultures from wild-type and D3R-KO mice. Primary cultures of mouse mesencephalic neurons form wild-type and D3R-KO mice were stimulated with nicotine (Nic; 10 μM) or quinpirole (Qui; 10 μM) for different times (0–14 h) and analyzed for p70S6K phosphorylation (p-p70S6K), CREB phosphorylation (pCREB), or c-Fos levels by western blot. **A** Representative blot of p-p70S6K, pCREB, and c-Fos. **B**–**D** Densitometric analysis of blots (*n* = 3) with specific levels of p-p70S6K (**B**), pCREB (**C**), and c-Fos (**D**) normalized to the corresponding TH and tubulin levels. Data were statistically analyzed by one-way ANOVA followed by post hoc comparison with Bonferroni test. (***p < 0.001, **p < 0.01, *p < 0.05 vs 0; post hoc Bonferroni’s test)

Previous observations have shown that the neurotrophic effects of nicotine require the PI3K-mammalian target of rapamycin (mTOR) signaling [6]. Therefore, we investigated whether the p70 ribosomal S6 kinase (p70S6K), a preferred substrate of the mTOR multiprotein complexes (mTORC1), that is, in turn, a cytosolic target of ERK1/2, is part of the signaling cascade activated by the D3R-nAChR heteromer [8, 9]. Neuronal cultures from wild-type mice, treated with nicotine or quinpirole (both at 10 μM; 5 min–14 h) and cultures from D3R-KO mice, treated with nicotine (10 μM; 5 min–14 h) were lysed and analyzed for p70S6K, phosphorylated at Thr 389 (p-p70S6K) by western blot. As shown in Fig. 6 (panels A and B), nicotine-induced p70S6K phosphorylation in a time-dependent manner, starting from 5 min, reaching the statistical significance between 30 and 60 min and returning to basal levels after 4 h of stimulation. Similar results were obtained in quinpirole-treated wild-type neuronal cultures (Fig. 6, panels A and B). By contrast, in D3R-KO cultures, incubation with nicotine did not increase the levels of p-p70S6K (Fig. 6, panel A and B), suggesting a unique role of the D3R-nAChR heteromer in this effect.

Among the nuclear ERK1/2 targets, the cyclic AMP responsive element-binding protein (CREB) transcription factor, phosphorylated at ser 133 (pCREB) [21], and the immediate early gene c-Fos, [7] were analyzed (Fig. 6). Neuronal cultures from wild-type mice were treated with either nicotine or quinpirole (both at 10 μM) for different times (5 min–14 h) and cultures from D3R-KO mice were treated with nicotine (10 μM; 5 min–14 h). Untreated and treated neurons were analyzed for pCREB and c-Fos levels by western blot. As reported in Fig. 6 (panels A and C), in wild-type cultures, both nicotine and quinpirole increased pCREB levels, an effect starting at 30 min of stimulation and lasting until 4 h (Fig. 6, panel C). In neuronal cultures derived from D3R-KO mice nicotine also significantly increased pCREB levels, reaching a statistical significance from 5 min to 1 h upon stimulation (Fig. 6, panel C).

Analysis of c-Fos expression was also performed. As shown in Fig. 6 (panel A and D), in wild-type cultures incubated with either nicotine or quinpirole, c-Fos expression was increased with significantly higher levels measured between 30 min and 4 h from stimulation. By contrast, in neuronal cultures derived from D3R-KO mice, c-Fos expression did not change in response to nicotine stimulation (panel D). Together, these results suggest that, in contrast with CREB phosphorylation, the increase of c-Fos levels is likely due to the activation of the D3R-nAChR heteromer.

### D3R-nAChR Heteromer-Induced PI3K-Akt Activation

**Fig. 7:**
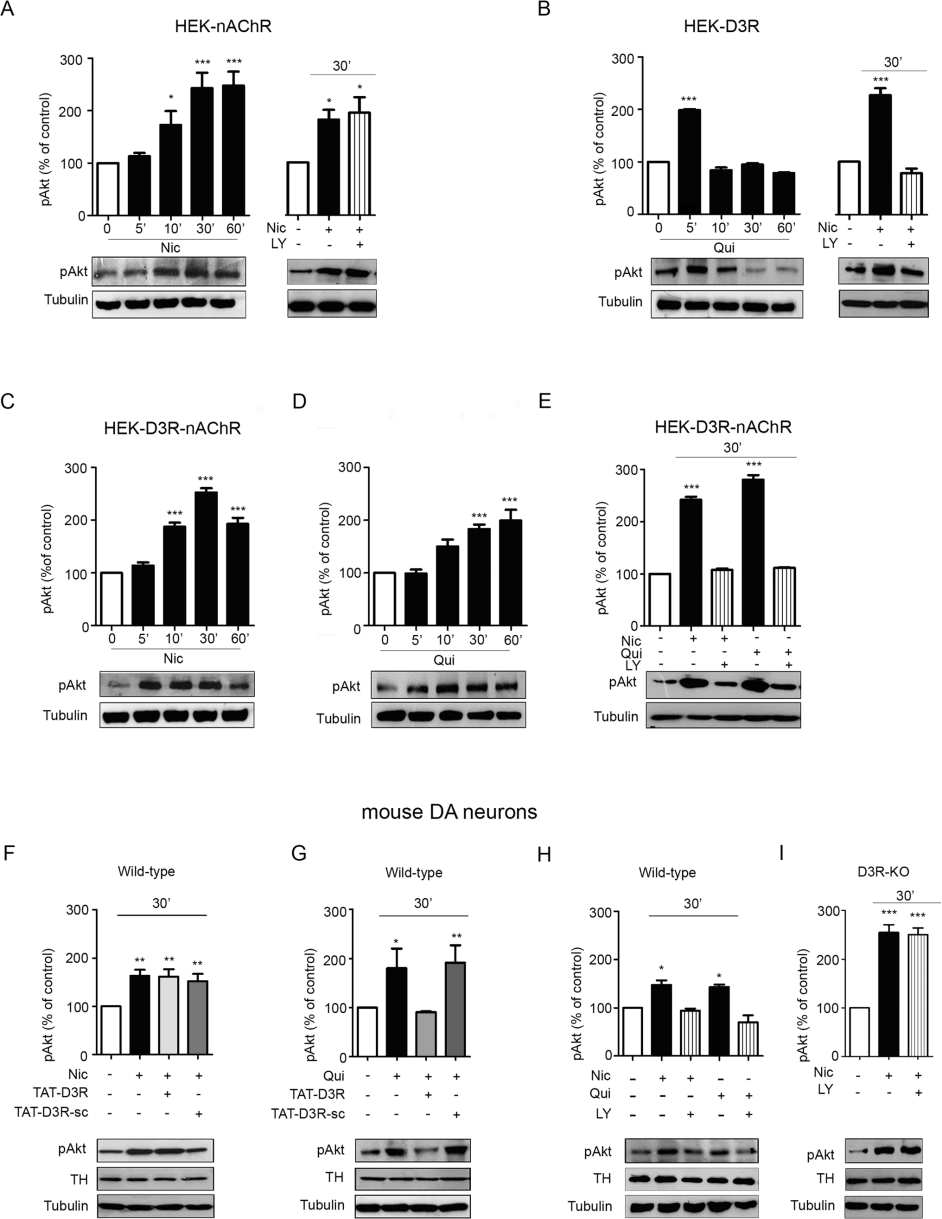
Analysis of D3R-nAChR heteromer-induced PI3K-Akt activation in HEK293T cells and in mouse neuronal cultures. **A** HEK293T cells transiently expressing the alpha4beta2 nAChR subunit (HEK-nAChR) were stimulated with nicotine (Nic; 10 μM) for different times (5–60 min) and analyzed for Akt phosphorylation at Thr308 (pAkt) by western blot; moreover, HEK-nAChR cells were stimulated with nicotine (10 μM) for 30 min in the presence or in the absence of LY294002 (LY; 10 μM) and analyzed for pAkt. Upper panel, densitometric analysis of blots (*n* = 3) with specific levels of pAkt normalized to the corresponding tubulin level. Lower panel, representative blot (image) of pAkt analysis; **B** HEK293T cells transiently expressing the D3R (HEK-D3R) were stimulated with quinpirole (Qui, 10 μM) for different times (5–60 min) and analyzed for pAkt by western blot; moreover, HEK-D3R cells were stimulated with quinpirole (10 μM) for 30 min in the presence or in the absence of LY294002 (10 μM) and analyzed for pAkt. Upper panel, densitometric analysis of blots (*n* = 3) with specific levels of pAkt normalized to the corresponding tubulin level. Lower panel, representative blot of pAkt analyses. **C**–**D** HEK-D3R-nAChR were stimulated with nicotine (10 μM) (**C**) or quinpirole (10 μM) (**D**) for different times (5–60 min). Upper panel, densitometric analysis of blots (*n* = 3) with specific levels of pAkt normalized to the corresponding tubulin level. Lower panel, representative Akt phosphorylation analyses by western blot. **E** HEK-D3R-nAChR cells were stimulated with nicotine (10 μM) or quinpirole (10 μM) for 30 min in the presence or in the absence of LY294002 (10 μM) and analyzed for pAkt. Upper panel, densitometric analysis of blots (*n* = 3) with specific levels of pAkt normalized to the corresponding tubulin levels. Lower panel, representative image of pAkt. **F**–**G** Primary cultures of mouse mesencephalic neurons were exposed to nicotine (10 μM) (**F**) or quinpirole (10 μM) (**G**) for 30 min in the presence or in the absence of TAT-D3R (1 μM) or TAT-D3R-sc (1 μM) and analyzed for pAkt by western blot. Upper panel, densitometric analysis of blots (*n* = 3) with specific levels of pAkt normalized to the corresponding TH and tubulin levels. Lower panel, representative blot of pAkt. **H** Primary cultures of mouse mesencephalic neurons were exposed to nicotine (10 μM) or quinpirole (10 μM) for 30 min in the presence or in the absence of LY294002 (LY; 10 μM) and analyzed for pAkt. Upper panel, densitometric analysis of blots (*n* = 3) with specific levels of pAkt normalized to the corresponding TH and tubulin levels. Lower panel, representative image of pAkt. **I** Primary cultures of mouse mesencephalic neurons derived from D3R-KO mice were exposed to nicotine (10 μM) for 30 min in the presence or in the absence of LY294002 (10 μM) and analyzed for pAkt. Upper panel, densitometric analysis of blots (*n* = 3) with specific levels of pAkt normalized to the corresponding TH and tubulin levels. Lower panel, representative image of pAkt. Data were statistically analyzed by one-way ANOVA followed by post hoc comparison with Bonferroni test. (***p < 0.001, **p < 0.01, *p < 0.05 vs untreated; post hoc Bonferroni’s test)

PI3K, crucially involved in the activation of the Akt/mTOR pathway and likely associated with D3R activity, has been shown to be crucial for nicotine-induced DA neuron structural plasticity [6]. In order to investigate the ability of D3R-nAChR heteromer in activating Akt, both HEK-transfected cells and mouse mesencephalic neurons were used. As shown in Fig. 7, analyses of Akt phosphorylation at Thr 308 (pAkt) by western blot indicated that HEK-nAChR cells, treated with nicotine (10 μM) for 5–60 min, induced a persistent activation of Akt (Fig. 7, panel A). Moreover, stimulating HEK-nAChR cells with nicotine (10 μM) for 30 min in the presence or in the absence of LY294002 (10 μM) did not impact on pAkt activation, thus excluding a role of PI3K activity in mediating this signal. As shown for Erk1/2, HEK-nAChR cells incubated with quinpirole (10 μM; 5–60 min) were unable to induce Akt (data not shown). Analyses of pAkt in HEK-D3R cells, treated with quinpirole (10 μM; 5–60 min), showed that stimulation of the D3R transiently activated pAkt (peak at 5 min), and that this activation was prevented by treatment with LY294002 (10 μM) (Fig. 7, panel B). The Akt pathway was then analyzed in HEK-D3R-nAChR cells, incubated with nicotine (10 μM) or quinpirole (10 μM) for 5–60 min showing the ability of both the treatments to induce persistent activation of Akt (Fig. 7, panels C and D); moreover, nicotine- and quinpirole-induced activation of Akt was abolished by co-incubating cells with each agonist in combination with LY294002 (10 μM; 30 min) (Fig. 7, panel E). The D3R-nAChR heteromer-induced persistent activation of the PI3K-Akt pathway was then verified in primary cultures of mouse mesencephalic neurons derived from wild-type mice and D3R-KO mice (Fig. 7, panels F–I). We found that in wild-type cultures, Akt was still active after 30 min of both nicotine (10 μM) and quinpirole (10 μM) stimulation (Fig. 7, panels F and G). Moreover, as shown for pErk1/2, in wild-type neurons treated with nicotine (10 μM; 30 min), the presence of the TAT-D3R peptide, as of the TAT-D3R-sc (both at 1 μM), led to persistent phosphorylation of Akt; by contrast when neurons were treated with quinpirole (10 μM; 30 min), disrupting the D3R-nAChR heteromer by the means of the TAT-D3R peptide, but not the scramble one, counteracted Akt pathway activation (Fig. 7, panels F and G). Finally, treating wild-type cultures with nicotine or quinpirole (both at 10 μM; 30 min) in the presence of the PI3K inhibitor LY294002 (10 μm) significantly decreased pAkt levels (Fig. 7, panel H). Interestingly, in neurons expressing the only nAChR and derived from D3R-KO mice, nicotine treatment in combination with LY294002 (both at 10 μM; 30 min) did not affect Akt phosphorylation (Fig. 7, panel I).

Together, these data strongly indicate that in DA neurons, the activation of the D3R-nAChR heteromer results in the persistent activation of the PI3K-Akt pathway. Of note, as found for Erk1/2, while nAChR were equally able to activate the Akt pathway in a persistent way, it is likely that the PI3K activity is not required.

## Discussion

The property of D3R of inducing neurite outgrowth and increasing dendritic arborization and soma size in DA neurons has been previously reported both in vivo in rat models [15, 17] and in mouse and human in vitro cell models [5, 13, 16, 22, 23]. These events represent a fundamental form of neuroplasticity, the structural plasticity, in which neurons reorganize their structure and connections. Changes in neuron morphology occur over time and in response to alterations in the cell environment that act by engaging molecular mechanisms that are critical for cell growth and survival and that impact on the cell morphology [24]. Structural plasticity is also commonly observed after chronic exposure to all drugs of abuse [25–27]. Among them, nicotine, by activating beta2-containing nAChR expressed on DA neurons, increases dendritic arborization and soma size of these neuron population [6, 13], an effect sustained by the simultaneous activation of the D3R [5, 6, 22]. Interestingly, the functional cooperation between nAChR and D3R is related to the formation of a heteromeric receptor complex (the D3R-nAChR heteromer) through a physical interaction between the third intracellular loop of the D3R and the nAChR beta2 subunit [3]. Thus, the D3R-nAChR heteromer represents the molecular unit that is expressed on DA neurons, is activated by nicotine and D3R agonists, and is associated with the specific function of promoting neurotrophic effects. A PI3K-dependent signaling pathway, likely associated with the D3R, has been shown to be specifically responsible for this effect [3, 6, 13].

It is now clear that most G protein-coupled receptors (GPCRs) exist as homo/heterodimers [28, 29]. In particular, GPCR heterodimers are defined as single entities formed by two different protomers that are likely equally essential in inducing a given function [29]; as novel receptor entities, their specific distribution in addition to their pharmacological, signaling and trafficking features may be defined [29]. Previous data suggested that D3R stimulation possibly exerts neurotrophic effects “per se” [5, 13], while alpha4beta2 nAChR gains the ability to induce structural plasticity on DA neurons only when it is coupled to D3R. In this study, we clearly show that, as the nAChR, also the D3R, when expressed as a monomer, does not elicit neurotrophic effects. In particular, using mouse and human DA neurons morphological remodeling as the index of heteromer activation, and by means of the TAT-D3R interfering peptide disrupting the heteromer [3, 4], we found that the chronic stimulation with the D2R/D3R agonist quinpirole significantly exerted remodeling properties, leading to DA neurons morphologically characterized by increased dendritic arborization and soma area. However, these effects were lost when the heteromer was disrupted with the TAT-D3R interfering peptide. Therefore, quinpirole-induced structural remodeling is associated with activation of D3R assembled into the D3R-nAChR heteromeric complex; moreover, in these experiments in which the D3R interaction with nAChR was counteracted, both the monomeric D3R and nAChR, as well as D2R, were functionally able to trigger theirs signaling pathway. However, as described, quinpirole was unable to induce morphological changes, thus indicating that not only the D3R but also the D2R are not involved in these effects [5; 22]. Therefore, although quinpirole is a D2R/D3R agonist, a functional selectivity in inducing neurotrophic effects is related to the only stimulation of D3R-containing heteromer that represents the smallest molecular entity required for this type of response. Interestingly, in both mouse and human DA neurons, combining nicotine and quinpirole at low doses, that are ineffective in exerting neurotrophic effects, was sufficient for inducing structural plasticity thus suggesting that D3R-nAChR heteromer activation in DA neurons is ensured even under conditions of low neurotransmitter levels. It is important to note that among the different DA receptors, D3R display the highest affinity for DA [30–32] that is an additional warranty for this heteromer to be activated.

As a new entity, the D3R-nAChR heteromer was investigated for the signaling pathway associated with its stimulation. A large body of evidence has accumulated showing that when a GPCR heterodimer is formed, its peculiar signaling pathway could result from several mechanisms leading to potentiation or attenuation of a defined signaling pathway associated with one or the other protomer [28, 33, 34] or changes in G-protein coupling conferring to the receptor complex a different and unique signaling mechanism [35–37]. Previous observations in both mouse and human DA neurons have shown that the engagement of the PI3K, leading to the transient activation of ERK and Akt/mTOR likely associated with the D3R, is required for nicotine-induced DA neuron structural plasticity [6, 13, 23, 24]. Therefore, it is likely that this intracellular signaling could be associated with heteromer stimulation and strictly related to its ability in exerting neurotrophic effects.

PI3K belongs to a family of heterodimeric lipid kinases and its activity induces numerous cellular responses including development, growth, plasticity, and survival [38–41] by the recruitment of a variety of downstream effectors [42]. Among them, the ERK1/2 cascade is a fundamental converging pathway with distinct cell fate decisions; interestingly, the kinetic of ERK1/2 activation, such as duration, localization, or strength, dictates the discrete phenotypic responses [7]. On these bases, transfected HEK293 cells were used to analyse the dynamic of ERK1/2 activation and its relationship with PI3K activity associated with D3R-nAChR heteromer stimulation and compared to those activated by nAChR and D3R, individually expressed. We found that while stimulation of the D3R induced a rapid and transient activation of ERK1/2 that strongly depends on PI3K activity, stimulation of nAChR leads to a persistent increase of activated ERK1/2 that does not require PI3K activity; in cells expressing both D3R and nAChR that are associated into the D3R-nAChR heteromer [3; this study], stimulation with nicotine or quinpirole induced a PI3K-dependent sustained activation of ERK1/2. Persistent activation of ERK1/2 in response to nicotine or quinpirole was also observed in wild-type mouse primary neurons expressing both D3R and nAChR. Interestingly, ERK1/2 activation was significantly impaired by both the TAT-D3 interfering peptide and the PI3K inhibitor. By contrast, in primary neurons derived from D3R-KO mice, that only express nAChR, nicotine treatment induced a persistent activation of ERK1/2 that did not require PI3K activation. Therefore, a long-lasting activation of ERK1/2 as a downstream effector of PI3K is a peculiar molecular characteristic of the D3R-nAChR heteromer signaling pathway.

The subcellular distribution of active ERK1/2 induced by the heteromer or by nAChR was analyzed by using immunofluorescence. The results show that in DA neurons from wild-type mice, nicotine and quinpirole induced the activation of ERK1/2 that were localized both at cytoplasmic and nuclei sites. Similar results were obtained in DA neurons from D3R-KO mice following nAChR stimulation by nicotine, thus suggesting that the ability to module both cytoplasmic and nuclear targets is a common property shared by both nAChR and D3R-nAChR heteromer. Interestingly, when analyzing the total amount of active ERK1/2, we found that levels activated by the D3R-nAChR heteromer were significantly higher than those activated by the nAChR. This is relevant since evidence has been provided that not only signal duration but also total amplitude of ERK activity dictates a specific biological outcome [7, 43]. Therefore, a quantitatively high level of active ERK1/2 is a further specific characteristic of the D3R-nAChR heteromer-induced ERK activation.

In order to define the D3R-nAChR heteromer-induced downstream targets, modulation of the Cyclic AMP responsive element-binding protein (CREB) by phosphorylation at ser 133 (pCREB) [21], and the expression of the immediate-early gene c-Fos, [7] were analyzed.

CREB is a transcription factor that, by binding to different target genes, is crucially involved in the plasticity and survival of DA neurons [21, 44] with a relevant role in the mechanisms underlying drug addiction [45–47]. Analysis of pCREB in mouse neurons expressing D3R and nAChR and treated with nicotine or quinpirole has shown a persistent activation of CREB, with a pattern of activation similar to that of ERK1/2. However, similar results were obtained in mouse neurons only expressing nAChR and treated with nicotine, thus indicating that phosphorylation of CREB is not relevant for DA neuron structural remodeling. In line with this observation, in DA neurons of the nucleus accumbens, dendritic complexity has been found to be reduced or increased by opiates and psychostimulants, respectively, even if in both conditions, the induction of CREB activity was observed [25].

c-Fos, is a transcription factor, that together with c-Jun, comprises one form of the AP1 transcription factors, important regulators of early transcriptional process evoked by cellular stimulation [48]. It is well known that c-Fos is a very unstable factor and its expression can vary from a few minutes to several hours after stimulation; these temporal changes are regulated by a combined action of protein kinases and proteases since, without phosphorylation, both c-Fos mRNA and protein undergo rapid degradation; by contrast, phosphorylation at ser 374 and ser 362 in the C-terminus is sufficient to reduce c-Fos degradation to some extent, thus allowing accumulation into the nuclei and, its sustained promoter activity [8, 49]. Intriguingly, c-Fos phosphorylation requires active nuclear ERK1/2, which occurs only upon a strong and sustained stimulation of ERK cascade [49–51]. Analyses of c-Fos in mouse neuronal cultures have shown that c-Fos levels were significantly increased in primary neurons expressing both D3R and nAChR and treated with either nicotine or quinpirole (with significant levels measured between 30 min and 4 h from stimuli), but not in neuronal cultures only expressing the nAChR. In line with the idea that c-Fos is a crucial factor for transducing short-term stimuli into long-term responses, while both nAChR and D3R-nAChR heteromer stimulation results in sustained activation of ERK1/2, only the heteromer induces c-Fos expression that, therefore, could represent a key molecular signal for D3R-nAChR heteromer-evoked DA neuron structural plasticity.

The involvement of the p70 ribosomal S6 kinase (p70S6K) in the molecular events underlying nicotine-induced remodeling has been previously described [22]. p70S6K, a preferred substrate of the protein mTOR Complex 1 (mTORC1), through its relationship with PI3K and Akt is involved in neuronal cell regeneration through stem cell renewal and is an essential neuroprotective pathway that controls critical events such as apoptosis, autophagy, and necroptosis [52]. Interestingly, p70S6K is a cytosolic target of ERK1/2 [8]; hence, the ability of the D3R-nAChR heteromer in phosphorylating and activating this kinase was analyzed. We found that in mouse neurons expressing both nAChR and D3R, but not in neurons only expressing the nAChR, both nicotine and quinpirole induced a long-lasting p70S6K phosphorylation at Thr 389, with a temporal pattern which reflects that of ERK1/2, thus supporting its downstream position in this signaling cascade; therefore, only in the presence of D3R, nAChR stimulation is associated with the ability to activate the p70S6K.

Together, these data indicate that both c-Fos and p70S6K are key molecular signals for D3R-nAChR heteromer-induced DA neuron structural plasticity, associated with the Erk1/2 pathway; intriguingly, not only p70S6K but also c-Fos has been shown to be coupled with the PI3K-Akt pathway [53–55], thus indicating a molecular convergence of the two PI3K-dependent signals, Erk1/2 and Akt. Moreover, a cross-talk between the two signaling pathways has been described, with the possibility of compensatory mechanisms through which the two pathways regulate each other, thus ensuring D3R-nAChR heteromer optimal activity [9; 56]. The ability of the D3R-nAChR heteromer in activating the Akt pathway, previously associated with nicotine-induced structural plasticity [6], was analyzed using both transfected cells and mouse neurons. As shown for Erk1/2, while D3R stimulation resulted in rapid and transient activation of Akt, in a PI3K-dependent way, stimulation of both nAChR and D3R-nAChR heteromer induced a persistent activation of Akt, with a mechanism that is independent or dependent by the PI3K activity, respectively. Therefore, the ability to activate Erk1/2 and Akt in a persistent and PI3K-dependent way is peculiar to the D3R-nAChR heteromer and associated with heteromer capability in exerting neurotrophic effects. By contrast, our data likely suggest that alfa4-beta2 nAchR-induced activation of persistent Erk1/2 and Akt does not require the PI3K activity. On this line, while the alfa7 nAChR have been clearly described as able to signal through a PI3K-dependent way, for example, in supporting nicotine-induced neuroprotective effects, for the alfa4beta2-containing nAChR, a direct involvement of the PI3K has not been evidently found [56, 57]. Moreover, similarly to that observed for Erk1/2, the ability of D3R in activating Akt/mTORC1pathway in a transient way has been previously reported [58, 59]; more in general, in vitro and in vivo studies have suggested that activation of D2-type DA receptors rapidly activates Akt-dependent pathways, while prolonged stimulation of D2-type receptors (over 30–60 min) decreases Akt phosphorylation, specifically at Thr308 [60–64]. Therefore, while the D3R activation is usually associated with transient phosphorylation of intracellular signals, it is likely that heterodimerization with nAChR keeps the D3R-mediated ERK1/2 and Akt/mTORC/p/70S6K pathways in a prolonged and strengthened active state. Modifications in the desensitization mechanisms of D3R, such as reduced G protein uncoupling, or reduced trafficking of both interacting protomers from membrane to intracellular sites, could explain the D3R temporal changes in activating their signals when the receptor interacts with the nAChR within the D3R-nAChR heteromer; on this line, evidence showing changes in the spatio-temporal dynamic of signaling pathways activated by a GPCR as a result of receptor heterodimerization have been provided [65] and are in line with the idea that allosteric modulation between the two protomers within a dimer strongly influences the proper characteristic of a GPCR heterodimer [2, 66, 67].

As described above, the PI3K pathway has been implicated in neuroprotection, being a strong survival pathway [68–73]. Intriguingly, strategies able to enhance the PI3K pathway have been associated with decreased injury to dopaminergic neurons both in in vivo and in vitro models [44, 68] and defective PI3K signaling has been observed in DA neurons of PD patients as well as in animal models of PD [68, 74]. On this line, we recently provided evidence that the D3R-nAChR heteromer is also associated with DA neurons neuroprotection [4]. In both mouse and human DA neurons, in fact, both nicotine and D3R agonists counteracted the accumulation of alpha-syn induced by glucose-deprivation as well as the morphological defects induced by the formation of alpha-syn aggregates; remarkably, neuroprotection induced by nicotine and quinpirole through D3R-nAChR heteromer activation was dependent on PI3K [4].

**Fig. 8:**
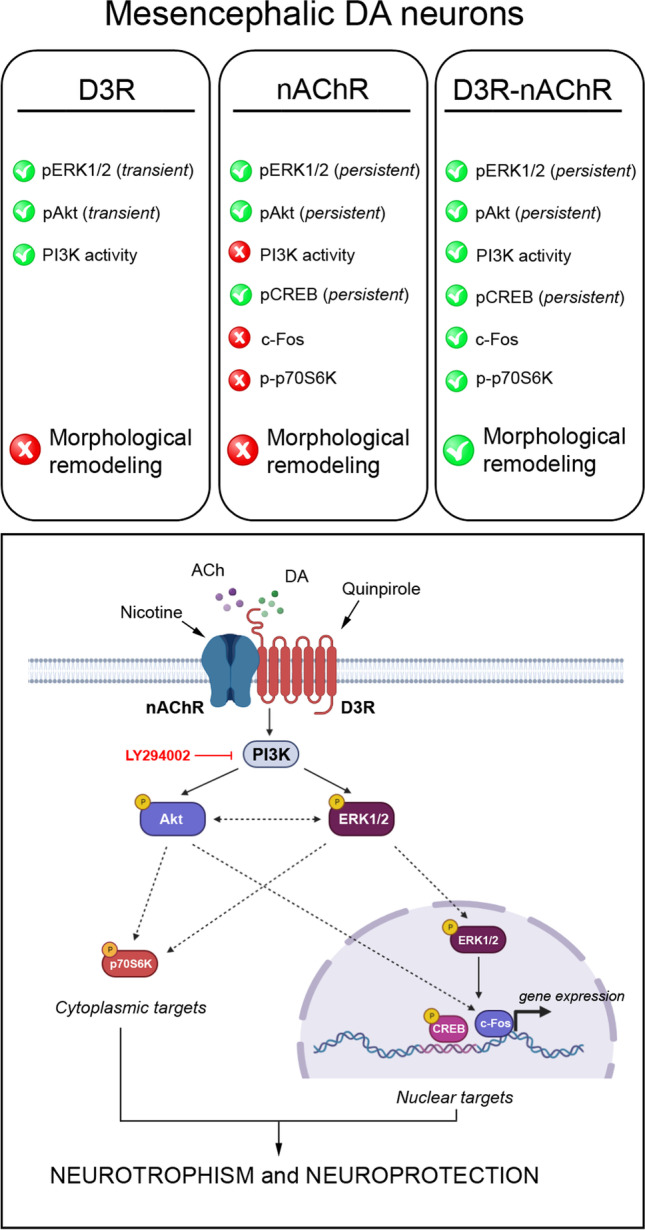
The D3R-nAChR heteromer transductional signaling pathway in mesencephalic DA neurons. Representative cartoon showing differences in the main intracellular pathway associated with morphological remodeling and activated by the single protomers or by the D3R-nAChR heteromer in mesencephalic DA neurons

Together, the data of this study suggest that in DA neurons, the D3R-nAChR heteromer signals through a PI3K-dependent pathway, likely coupled to the D3R protomer, with the expression of c-Fos and a sustained phosphorylation of p70S6K as key molecular signals critical for dendritic remodeling and for neuroprotection (Fig. 8) [4]. This heteromer may thus represent a specific target for compounds able to sustain and protect DA neurons [14]. In addition, an altered expression or function of this heteromer could critically impact on DA neurons health. On this line, we have recently characterized human IPSCs-derived DA neurons from two PD patients carrying the G2019S LRRK2 mutation. The major characteristics of these neurons are morphological alterations and spontaneous increase of non-fibrillary alpha-syn aggregates, likely directly associated with the mutation. Interestingly, DA neurons carrying the LRRK2 mutation were also characterized by reduced levels of D3R-nAChR heteromer at the plasma membrane [75] thus indicating that in these neurons, the possibility to activate the PI3K survival pathway, at least by the heteromer, is totally compromised. Affecting heteromer activity, as observed in LRRK2 G2019S DA neurons, possibly leading to reduced activation of PI3K-Erk1/2/Akt survival pathway may be a crucial molecular abnormality leading to DA neuron’s vulnerability.

## Data Availability

The datasets during and analyzed during the current study are available from the corresponding author on reasonable request.
